# Solutions to bridge the theory-practice gap in nursing education in the UAE: a qualitative study

**DOI:** 10.1186/s12909-021-02919-x

**Published:** 2021-09-13

**Authors:** Ahmad Saifan, Briliya Devadas, Fares Daradkeh, Hadya Abdel-Fattah, Mohannad Aljabery, Lintu Maria Michael

**Affiliations:** 1 Nursing College, Applied Science Private University, Amman, Jordan; 2 Nursing Department, Fatima College of Health Sciences, Abu Dhabi, UAE; 3 Department of Nursing, Fatima College of Health Sciences, Al Ain, UAE; 4 Department of Nursing, Fatima College of Health Sciences, Al Dhafra, UAE

**Keywords:** Theory-practice gaps, Nursing education, Solutions and suggestions, UAE, Qualitative

## Abstract

**Background:**

The theoretical knowledge of nursing underpins the practice, while the practice environment determines the circumstances within which the theoretical knowledge is applied. The biggest challenge facing nursing as an academic field is the theory-practice gap, which is a universal issue in nursing**.** This study aimed to raise solutions to close the gap between theory and practice in nursing education through the eyes of nursing students in UAE.

**Methods:**

A qualitative descriptive approach was followed; whereby 25 Emirati nursing students were interviewed.

**Results:**

Two main themes are discussed in this study: ‘Clinical Culture Creation’ and ‘Curriculum Content Reformation’. The students suggested to decreased the loud and stress on their clinical educators. They also suggested creating synchronization between what is taught in classrooms and labs and what is offered in the clinical training. Moreover, some of the students expected to have more benefit if they get their clinical training in health institutions owned by their college. On the other side, many of the participants suggested to move from the integration system to the block system. Another interesting suggestion includes having the same college staff to teach the theory and the clinical. The final suggestion includes decreasing the paper work during clinical training.

**Conclusions:**

This study explored the solutions to bridge the theory-practice gap in nursing education in the UAE. The study has implications for nursing education and practice within the UAE and is imperative for graduating workplace ready professional nurses within the country.

## Background

Theory and practice are the twin essential components of nursing education. The theoretical knowledge of nursing underpins the practice, while the practice environment determines the circumstances within which the theoretical knowledge is applied. Nursing is a scientific profession based on applied research, guided by evidence-based theory, and numerous concepts within the profession have focused on the art of nursing and theoretical sciences. However, the biggest challenge facing nursing as an academic field is the theory-practice gap, which is a universal issue in nursing and midwifery. It has been explained as a “distancing of theoretical knowledge from the actual doing of practice” [1, 2], and it affects newly qualified registered nurses when they embark on clinical practice, as well as nursing students during clinical placements.

Though professional knowledge is introduced as theory in the classroom settings, it is within laboratory sessions that this knowledge is translated into practice in nursing education, and in clinical practice in hospital settings, these skills are transferred, to provide meaningful need-based care for patients. Thus, the theory-practice gap is most obvious in nursing education in laboratory sessions and clinical practice. This theory-practice gap is concerning as it primarily affects patient safety negatively, while concurrently contributing to an unsatisfactory clinical experience for the novice nursing student in the clinical environment.

Organizational culture and processes act as an important motor of the theory-practice gap, in addition to student and instructor factors in the teaching-learning environment. Several studies suggest that the reasons for the existence of the gap are multifactorial [3]. The frustrations and difficulties associated with the gap are experienced mainly by student nurses and newly qualified nurses, and can harm their socialization into the professional role, and subsequent professional development [4–7]. The reality shock is considered a major cause for low job satisfaction and high job attrition rates among newly qualified nurses [8, 9]. The theory-practice gap has also been cited as a contributory factor in medication errors [10, 11] and reduced physical assessment skills use among nurses [12]. The existence of the theory-practice gap can influence the quality of nursing care and patient outcomes.

Several studies highlight the theory-practice gap in nursing education worldwide and in the Middle East and North Africa (MENA) region [6, 13–16]. Most reviewed studies in this field focused on searching for whether the gap is present or not, while some studies considered possible reasons for it. However, solutions to decrease this gap, and to link theory to practice, are rarely discussed and studied [14, 17, 18]. The role of nursing academics in linking theory to practice is crucial in closing the theory-practice gap [6, 14, 19], while the role of preceptor to assist the nursing students in developing clinical skills and integration into the culture of the clinical area cannot be emphasized more [20].

Several international studies from UK, Ireland, Turkey and the US have identified the importance of collaboration between nursing instructors, nurses, nursing school and hospital managements as one of the most suggested means of bridging the gap [14, 15, 20, 21]. Several studies showed the importance of the availability of adequate number of well-trained academics and clinical preceptors. These studies identified other strategies to close the gap between theory and practice in nursing education such the presence of clear context-based curriculum and availability of standard clinical guidelines [6, 14, 18]. Eggertson [22], an expert Canadian nurse, stressed on the need for undergraduate nursing education that better equip nurses to assume leadership roles in inter-professional teams and support them in providing high-quality patient-centered care.

The nursing program, at the College of Health Sciences in the UAE, is a 4-year Baccalaureate degree program, having initial program accreditation from the Ministry of Higher Education for Scientific Research (MOHESR), and institutional accreditation from the UAE Commission of Advanced Accreditation (CAA). The current program study plan consists of 127 Credit hours; 98 credits of theory, 9 laboratory sessions and 27 clinical placement. Laboratory practice sessions are opportunities for students to practice hands-on clinical competencies and skills, albeit in a safe environment. Students in clinical placement are accompanied by clinical instructors with a ratio of eight to ten students assigned to one instructor. While in the clinical placement units, each student is paired with a nurse, as preceptor, who will demonstrate the nursing care provided to patients and provide opportunities for students to practice hands-on on real patients. At the end of the clinical placement, the clinical instructor will evaluate the students, with the preceptor feedback, based on competency checklists and the CAT (clinical assessment tool that students are already familiar with.

In the UAE, every year nursing student cohorts are graduated and start their professional careers in hospitals around the country and internationally. There is shortage in the number of nurses in the UAE and the surrounding countries such Sadi Arabia, Qatar and Oman. These countries require a large nursing taskforce and a ready to practice nursing graduates. This is a major concern for healthcare service managers, and highlights the need for immediate action to expedite the process of attracting and graduating ready to practice, local nurses that understand the local culture and context and remain in the system (23, 24). Therefore, it is essential that these nurses are well prepared upon graduation so they can handle the requirements of their new roles [23, 24]. Reviewing the body of literature showed that suggestions and interventions to close the theory-practice gap in nursing education were rarely discussed worldwide. Moreover, there are no study to discuss this subject in the UAE.

It was thought that the best people to suggest solutions to close this gap are the nursing students who went through this experience. Finding solutions to the theory-practice gap before entry to practice may reduce the difficulties experienced by the graduating participants, and increase their ability to integrate their knowledge appropriately in the practice setting. The current paper aims to raise possible solutions to close the gap between theory and practice in nursing education through the eyes of nursing students currently studying and experiencing this phenomenon in nursing education in UAE.

## Methodology

### Design

This study aims to explore the strategies that could reduce the theory-practice gap in nursing education within the UAE context. This study used a descriptive qualitative approach to explore solutions to bridge the theory-practice gap in nursing education in the UAE. According to Glesne [25] (p. 4), “qualitative research methods are used to understand phenomena from the perspectives of those involved, to contextualize issues in their particular socio-cultural-political milieu, and sometimes to transform or change social conditions.” Face-to-face qualitative interviews are commonly used due to their usefulness and the fact that a great amount of information can be ascertained by simply questioning people in an interpersonal format. Qualitative research attempts to increase our understanding of why things are the way they are in our social world, and why people act in the ways they do [26]. The following research questions were initially used to explore the solutions to the theory-practice gap in the UAE.

1. Tell us about your first experience in training in clinical settings. How can you describe this experience?
2. Did you find differences between what you have been taught in classrooms and what you have faced in the clinical training? Please, explain …
3. How did your mentor’s support and understanding of what you learned in theory help you in practice?
4. How do you think we could bridge the theory-practice gap in nursing education?

### Sample and sampling

Data for this study was collected from participants studying at a main College of Health Sciences in the UAE. This college has an accredited nursing program and have almost the biggest pool of nursing students and graduates in the country. Established in 2006, the College aims to meet the UAE’s growing need for skilled healthcare professionals. Being the first nursing college in the UAE to study participants’ experience in the clinical setting, the qualitative approach would help obtain rich and in-depth information. An interview schedule was developed based on the literature review, but an emergent design was used to enable the asking of probing and follow-up questions to explore emergent areas of interest [27].

Sampling is an essential step in the research process as it helps to inform the quality of interpretation made by the researchers that stem from the underlying results [28]. Purposive sampling technique was used to elicit participant views regarding the prevalence of theory-practice gap in nursing education in the UAE, from among the Nursing Program participants. The proposed participants for the study were selected based on specific criteria. They needed to be at the third-year level onwards as participants who had attended at least two or more clinical courses, because they would have a better understanding of the study topic under exploration and have had enough clinical practice to compare with the theoretical component. The College has branches in four emirates within the country, and participants were recruited from all four campuses. Bridging participants were excluded because of their prior work experience in hospitals, and the difficulty this could present to exploring the theory-practice gap specific to nursing education (as opposed to registered nurses’ practice).

A total of 25 participants expressed interest in sharing their views on the subject. As the College currently hosts only female participants within their population, 100% of the samples obtained were females. Both Emirati (local) participants and expat (non-national) participants were included in the study sample. According to Polit and Beck [27](p. 273), the “sample size in qualitative interviews is usually determined based on informational needs, and is approximately ten or until data saturation occurs”. Thus 25 participants in this study was more than sufficient to gain in-depth and rich data. Table 1 illustrates the demographic characteristics of the participants in this study.

**Table 1:** Demographic variables regarding the participants

Participant no.	Level of study	Age	Nationality	Emirate
1	BN4^a^	21–23	Expatriate	Al Dhafra (ADH)
2	BN4	21–23	Expatriate	Al Dhafra
3	BN4	21–23	Expatriate	Al Dhafra
4	BN4	21–23	Expatriate	Al Dhafra
5	BN4	21–23	Expatriate	Al Dhafra
6	BN4	21–23	Expatriate	Al Dhafra
7	BN4	21–23	Emirati	Ajman (AJ)
8	BN4	21–23	Emirati	Ajman
9	BN3	21–23	Emirati	Ajman
10	BN3	21–23	Emirati	Ajman
11	BN3	21–23	Emirati	Ajman
12	BN3	21–23	Emirati	Ajman
13	BN4	21–23	Emirati	Al Ain (AA)
14	BN3	21–23	Expatriate	Al Ain
15	BN3	20	Emirati	Abu Dhabi (AUH)
16	BN3	20	Emirati	Abu Dhabi
17	BN4	21–23	Emirati	Abu Dhabi
18	BN4	21–23	Emirati	Abu Dhabi
19	BN4	21–23	Expatriate	Abu Dhabi
20	BN4	21–23	Emirati	Ajman
21	BN4	21–23	Emirati	Ajman
22	BN3	20	Emirati	Al Ain
23	BN3	20	Emirati	Al Ain
24	BN3	20	Emirati	Al Ain
25	BN3	20	Emirati	Al Ain

^a^(BN3 and BN4 reflects the level of study; BN3 means year 3 and BN4 means year 4)

### Ethical considerations

Gerrish and Lacey [29] state that ethical implications should be addressed in every research stage, including protecting participants and researchers from harm, using voluntary participation, anonymity, and confidentiality, as well as informed consent. This study obtained ethical approval from the College Research Committee and College Institutional Review Board. All participants were given a participant information sheet and were made aware of their rights to withdraw from the study at any stage without giving a reason, and that this would not affect their educational progress or statutory rights. Anonymity was assured. All interviews obtained were audiotaped, and transcriptions were used for analysis purposes. All interviews were transcribed verbatim and were used only for this study. Participant confidentiality was maintained throughout. All methods were carried out **in** accordance with relevant guidelines and regulations.

### Data collection procedure

After receiving ethical approval, invitation letters were sent via email to the nursing participants using their College email addresses, explaining the nature and scope of the study and participation expectations and rights, and inviting them to participate. Participants were asked to contact the research corresponding author if they wished to take part. Contact information for the corresponding author was added to the email and participants were given the chance to ask any questions or for any clarification before making their mind whether to participate or not. When they agreed to participate in the study and met the inclusion criteria, arrangements for interviews were made. Two expert interviewers did all interviews. These interviewers met with the primary author and discussed the interview guide. Each interviewer conducted two pilot interviews and shared the results with the primary author, then another meeting was arranged between the primary author and the interviewers. This meeting aimed to unify the interviewing method and to have similar interviewing questions and conditions. The reason to select two interviewers was to avoid social desirability and other forms of bias in the interviews, by avoiding interviews being conducted by participants’ teachers. In other words, the interviewers did interviews for participants out of their campuses, and they had never met them before.

### Data analysis process

Computerized thematic analysis (NVIVo) was used to analyze the data that was generated by the interviews, similar to previous studies [30, 31]. It was decided to assign Braun and Clarke [31] technique of thematic analysis to deduce emerging themes. They identified six phases of thematic analysis (Table 2), and these steps were undertaken in the study. Some sections of the interviews were in Arabic, which is the participants’ predominant native language. Transcribers fluent in Arabic assisted in the transcription and translation, and translations were checked for content accuracy and validity with the interviewers before analysis.

**Table 2:** Phases of thematic analysis [27]

Phase	Description of the process
1. Familiarization with data	Transcribing data (if necessary), reading and rereading the data, noting down initial ideas.
2. Generating initial codes	Coding interesting data features in a systematic fashion across the entire data set, collating data relevant to each code.
3. Searching for themes	Collating codes into potential themes, gathering all data relevant to each potential theme.
4. Reviewing themes	Checking in the themes work in relation to the coded extracts (Level 1) and the entire data set (Level 2), generating a thematic “map” of the analysis.
5. Defining and naming themes	Ongoing analysis to refine the specifics of each theme and the overall story the analysis tells; generating clear definitions and names for each theme.
6. Producing the report:	The final opportunity for analysis. Selecting vivid, compelling extract examples, the final analysis of selected extracts, relating the analysis to the research question and literature, and producing a scholarly report of the analysis.

To build up the study themes, the researcher read the data repeatedly and highlighted common phrases to prepare the required codes. Interview extracts were highlighted and classified under possible codes. Participants’ phrases were highlighted with the same color to correspond to the codes, then the patterns among codes were identified to create the study sub-themes.

## Results

A total of 25 female full-time participants studying in the Bachelor of Nursing (BN) program at FCHS participated in the study. Their ages ranged from 21 to 23, and 17 participants were Emiratis, while 8 were expatriates. Eleven participants studied at the BN3 level, while the remaining 14 participants were at the BN4 level. Participants were distributed among the four FCHS campuses: Abu Dhabi (*n* = 5), Ajman (*n* = 8), Al Ain (*n* = 6), and Al Dhafra (n = 6). Nearly all the participants, directly or indirectly, made statements that reflected presence of a gap between what they have taught in the theory and what they have found in the clinical trainings. Many reasons were raised to explain the presence of this gap. Interestingly, most of the participants have raised some suggestions and solutions to close this gap directly after talking about these reasons. Therefore, it was important to focus on students’ suggestions and solutions to close the gap in nursing education in UAE. Four main themes are discussed in this study.

The students suggested decreasing the load and stress on their clinical educators. They also suggested creating synchronization between what is taught in classrooms and labs and what is offered in the clinical training. Moreover, some of the students expected to have more benefit if they get their clinical training in health institutions owned by their college. All these subcategories were collected under one major theme ‘Clinical Culture Creation’. On the other side, many of the participants suggested to move from the integration system (theory and clinical in the same time) to the block system (having the theory during the first half of the semester, and then having all the clinical trainings in the second half). Another interesting suggestion included having the same college staff to teach the theory and the clinical. This was expected to create more coordination between theory materials and clinical needs. Another suggestion was to decrease the paper work during clinical training. These suggestions have been grouped under another major theme ‘Curriculum Content Reformation’ (Table 3). Embedded within the above two themes, two other themes have also emerged – Learning by Reflection; and The instructors’ Approach. The themes have been elaborated below.

**Table 3:** Study Themes

Major Themes	Subthemes
1. Clinical Culture Creation	➢ Increased Clinical Loads on College Clinical Instructors ➢ Dis-Unification of Competencies ➢ Lack of Own Clinical Facilities
2. Curriculum Content Reformation	➢ Need for Adopting the Block Clinical Rotation system ➢ Lack of Congruence between Theory and Clinical Training Too Much Focus on Workbook and Clinical Grade
3. Learning by reflection	
4. The instructors’ approach	

### Clinical culture creation

Clinical training is a foundation of nursing practice and a salient feature in nursing literature. It is considered a basic block of nursing education, and this is exemplified in attempts to delineate and enrich the participants’ knowledge and skills throughout their program of study. All the participants have raised issues regarding the clinical training and they linked these issues with widening the gap between theory and practice in nursing education. The following discussion describe these issues under three subthemes.

#### Increase clinical loads on college clinical instructors

Participants revealed hesitation infrequently engaging with their instructors. Their reasons were related to the number of participants per group, the time designated to allow the participants to practice in each unit to understand all the nursing care required, and the clinical faculty instructor responsible for allocating more time to participants to bridge the gap between theory and the clinical training. As one of the participants reported:

“They are pressured, I have seen this in Aged Care clinical, Mr. X was very pressured, he had many groups, he tried his best to finish the requirements and to help us, and he did actually. However, if he was always available in the hospital, maybe we would have benefited more. the same thing I said earlier.” (P. 3, ADH Campus)

The students wanted to say that college clinical instructors should have more time to and fewer duties. This will facilitate their job and will enable them to spend more time with their students. They admitted the role of college clinical instructors to decrease the gap between theory and practice. Several students suggested assigning less number of students to each college clinical instructor. They explained that doing this will increase the time assigned to each student in the clinical practice and will allow the instructors to support their students.

“Oh … What I can say. They follow big number of students. They also have lots of paper work to finish. If the college assigned them to less number of students, they will be able to spend more time with us … They will be able to support us and to fill any gap in clinical education. I would suggest to assign each instructor to 3 or 4 students only.” (P15, AUH Campus)

#### Di-unification of competencies

According to the year of study, the competencies taught during the college lab sessions have to be twined with what the participants are exposed to during their clinical placement, in the clinical premises. Such similarity would provide participants with an increase in desire for clinical training and theoretical knowledge, and students will be more involved with patient care. According to their year of study, the competencies taught during the college lab sessions have to be twinned with what the participants are exposed to during their clinical placement in the clinical premises. Such similarity increases students’ desire for clinical training and learning theoretical knowledge, which consequently involves them more in patient care. Similarly, many participants mentioned that the same instructors supervising their clinical training is essential. Subsequently, the partnership between lab sessions and clinical instructors motivated the students in their clinical learning. Such a partnership approach has been emphasized in nursing literature for decades, and has continued to date [32, 33]. As one participant remarked:

“My suggestion is to reduce the clerking on the clinical teaching as they have too many competencies to be completed in each clinical course, which is overwhelming, and diverts our focus … All of us would be preoccupied in completing these competences before the deadlines, and this will keep us away from doing hands-on procedures and engagement with patients. At the same time, having the same instructor who teaches us the theory and/ or the lab session supervising our clinical training would facilitate our clinical learning.” (P. 11, AJ Campus)

A few number of students provided practical solutions to the above problems. They suggested summarizing all the competencies requested from the students in the practice training. They suggested to share these competencies with all college academics and to explain them to the students before starting their clinical training. These students suggested adding more flexibility to implement these competencies. In other words, the students can take any opportunity to implement the available competency during their clinical training. This was expected to decrease the pressure on students and to give them more time to fulfill all the required competencies.

“Honestly, we do not have time for hands-on training. Each clinical course has different competencies, and we are requested to implement all of them during the semester. Sometimes, we keep waiting for one of the competencies for long time … We do not get the opportunity to implement all competencies during the semester. This add more pressure on us and make us anxious. If we have flexibility in implementing these competencies, this will ease our duty and will give us more chances to focus on the available competencies”. (P. 22, AA Campus)

#### Lack of own clinical facilities

There are some students explained that many medical-related education fields have clinical entities, facilitating feelings of belongingness, loyalty, and accountability. Such feelings facilitate and empower participants and clinical educators, who can ease the clinical training for both the clinical facilitators and participants during their placements. As one participant mentioned:

“The gap, first of all, is the hospital; if we had our own hospital it would be very nice; maybe we could have the labs in the hospital also. Maybe there would be patients who come to the hospital to take the treatment, but like, for less than other hospitals. Therefore, patients would come to our FCHS hospital because it is less costly.” (P. 6, ADH Campus)

This suggestion was not raised earlier in the literature. The students wanted to obtain all clinical training in one place owned by the college. This might be a difficult request, as the nursing curriculum includes several and different clinical courses such as medical-surgical, pediatric, maternity, mental health and community. It will be challenging to have all these courses in one place. The students might want to obtain their clinical training in more comfortable placements. This might be clear when one of the students described the clinical placements as a strange place for her. She explained that the preceptors were not familiar with the college curriculum and the objectives of their clinical courses. She indicated that there were no communication between their college clinical instructors and the preceptors in the clinical placements.

“The problem … No one know who we are and what we were requested to do. They did not have any idea about our curriculum. I think that there is a need for more communication between our college and the staff in the hospitals.” (P. 21, AJ Campus)

It should be indicated that only one student talked about the above barrier to link theory and practice. However, this might be shared by other students in the college and may influence negatively on the clinical training.

### Curriculum content reformation

The second major them of the theory-practice gap concerned curriculum implementation in the classroom, which encompassed three sub-categories.

#### Need for adopting the block clinical rotation system

An area of reform revealed by participants related to the structure of the nursing programs’ teaching methodology. Participants felt that the nursing teaching plan could adopt the block model of clinical rotation rather than the integration model. This would help them complete and consolidate the theory of education first, improving their ability to translate their knowledge and abilities to the clinical practice through their clinical placement. Some participant views are shared below:

“I think we should finish the theory or clinical skills or laboratory skills; we should learn in the College first, and then we should start the clinical rotation. If we did not learn about the skills before and went to the clinical, it will be hard for us, because we do not know what it is and what to do. Therefore, if we already learned the skills in the laboratories then went to the clinical rotation, it would be easier for us and we will be familiar with the procedures done.” (P. 13, AA Campus)

One participant felt the need for clinical practice to inform the theoretical curriculum, contrary to the prevailing ethos of the curriculum and nursing education in general:

“Maybe we should focus on the clinical training or the clinical placement. We should link the clinical with the theory, not the theory with the clinical, because sometimes we cover a lot of things in the theory but it’s not relevant or current to the clinical practice.” (P. 20, AJ Campus)

It is important to indicate that the block system was adopted earlier in the college under study. The students used to finish the theory in the first half of the semester, and then they continue the clinical during the other half. The study plan was changed recently, and all the third and fourth year students went through a transitional stage of changing the study plan. The current study plan includes integration of theory and clinical. This means that the students study the theory and go to the clinical training every week during the semester.

#### Lack of congruence between theory and clinical training

Many participants related that teaching the theory of the subjects and clinical skills throughout the semester would improve their understanding of the course content. They reported that the educator would be better aware of the theory content covered and the clinical skills related, and would be able to merge these through the teaching and clinical training, as reflected in the following examples:

“I feel the one who is teaching should be the one with me in the clinical, because when it’s like this, I have a better experience in the clinical, but when it’s a different instructor, I feel like sometimes he becomes lost and I become lost.” (P. 13, AA Campus)

“So, when I reflect on my experiences in the clinical each semester, when it’s the same person who is teaching me, the experience is better. I get better experiences. I remember each day and what I do, but if I get a different instructor, I feel like he is lost and I am lost and I don’t get the same results as when it’s the same.” (P.24, AA Campus)

A few number of students suggested that college clinical instructors should have extensive clinical experience in the field of their specialty. They clarified that some college clinical instructors have excellent academic experiences and extensive theoretical knowledge about the courses they teach. However, they do not have good clinical experience or they never been in the clinical placements before. These instructors may have short experience or have not any experience as nurses in the field. These instructors were unable to support the students in the clinical training.

“I will tell you an example. One of our academics was amazing in the theory class, but she was not confident in the clinical. I think that she never worked as a nurse before. I noticed that she became stressful when she taught us in the clinical. It will be great if they keep her in theory and assign another instructor with more clinical experience.” (P. 1, ADH Campus)

#### Too much focus on workbook and clinical grades

The participants’ aim and focus during their clinical placement was primarily to complete the clinical workbook as a requirement for clinical assessments and grades, rather than acquiring skills and knowledge per se. This diverted their focus from clinical training and gaining skills due to the overriding objective of meeting their grade requirement for many participants. By focusing on filling the needed component of the workbook, time spent engaging with preceptors and being involved in clinical practice was limited within the course’s clinical component. Many students said they would prefer a unified way of teaching, combining theory/lab and clinical training, as such learning would facilitate their clinical training, minimize negative impressions from nurses, and enable more hands-on education, leading to motivation in student learning:

“Instructors usually give examples from their experiences while explaining concepts in the theory or the lab sessions, and such simulation will be very effective if they also continue teaching and supervising during the clinical placements.” (P.18, AUH Campus)

“Such unification between what we are taught in the College and what we are exposed to in the hospitals would make us feel that what we learn is what the nurses do in clinical practice, and make it easier for us to engage and involve with procedure and patients.” (P. 25, AA Campus)

Most of the students suggested decreasing the load on students in the clinical placement. They indicated that the heavy paper works shifted their focus and decreased their chances to improve their clinical skills. Some of these students went further to describe the clinical placement as similar to theory classes with only one difference, which was the place. One of the fourth year students said:

“They keep us always busy with paper works. I spend more time in writing and preparing in the clinical placement more that the theory classes. They have to give us more space to see real cases and to get more skills.” (P. 17, AUH Campus)

### Learning by reflection

A lot of the student found it important to practice reflection in a clinical setting. Many students explained that reflection enhanced their learning through the retention of information. Reflection contributes to boosting students’ clinical skills as it helps in correcting mistakes and in feeling more confident when providing patient care. In addition, four students reported improvements in their analytic skills. For example, one participant noted that:

“Reflection provided me with the opportunity to evaluate my performance”. (P. 7, AJ Campus)

Some of the students preferred to interact with the clinical preceptors and the nurses during the clinical training. They thought that this would present them to the real world of nursing. They suggested to assign an hour at the end of each clinical training shift to discuss all what they have learned during the day with their college instructor.

“Instead of spending the time with our academics in the clinical placement, we can learn more about the real nursing world by the preceptors. We can discuss everything we have learned at the end of each clinical day.” (P. 11, AJ Campus)

### The instructors’ approach

Many students regarded their instructors’ support as essential for reflection, especially when the instructors provided them with guidance and feedback on their performance. They highly valued instructors who were passionate, considerate and cooperative. The type of questions asked by their instructor guided them to look for information and helped to improve their analytic skills. One students explained that:

“Some instructors are very friendly and helpful. They are always keen to answer my questions, address my concerns, and acknowledge my work. These actions motivated me to improve my skills by reflecting on my experience.” (P. 14, AA Campus)

In summary, the findings of the current study have raised several interventions and suggestions to bridge the theory-practice gap in nursing education. Some of these interventions focused on improving the culture of clinical training, while other suggestions shed some light on revisiting the curriculum and make some changes to decrease the loads on students during clinical training. Finally, the findings showed the importance of having good reflection at the end of each clinical training day and providing support to the students by their college instructors.

## Discussion

The study has revealed some possible solutions to the theory-practice gap from the students’ perspectives. Within the clinical arena, participants revealed that the clinical instructor played a vital part in optimizing student-learning experiences. A similar finding was observed by Ahmed, Ahmed [34] among nursing participants in Egypt, where participants revealed that the quality of student-clinical teacher interaction could either facilitate or hinder participants’ learning experiences. Needham, McMurray [18] and McSharry and Lathlean [20] stressed that active collaboration between the clinical nursing instructor and the nursing preceptor facilitated student learning positively. In another qualitative study conducted among undergraduate students in Jordan, students voiced that they did not receive good support in the clinical area, with some explaining that their instructors were sometimes busy in other managerial issues, decreasing the time left teaching for students [16]. The disparity in ratio between instructor and students was also highlighted in a study by Shoghi, Sajadi [21], where it was revealed that nurse-to-nurse faculty/clinician ratio increased with large admissions, and consequently reduced the value and extent of learning experiences in the clinical environment.

Participants voiced discontent regarding the differences between procedures taught and those observed on clinical placements concerning the nursing competencies. In a recent paper by Billings [35] and Huston, Phillips [15], they stressed that at the curriculum level, faculty must consider the national nursing competencies as a curriculum thread and plan learning activities and clinical practice assignments to implement them within all courses. Tanriverdi, Ozyazicioglu [36] conducted a study in Turkey among the third- and fourth-year undergraduate nursing students and revealed that providing parallelism between the practicing field and the students’ needs was crucial to optimal learning. Their study also revealed that students preferred an integrated model of clinical practice, because it seemed to reduce the gap between the classroom and the clinical area. This was contrary to the findings that emerged from the current study, where students shared a preference for the block system of clinical rotation, on the basis that completion of the didactic content first would provide much-needed familiarity and confidence in their clinical practice.

There is a growing body of knowledge regarding the use of clinical simulation in nursing education to bridge the gap between theory and practice [2, 13, 37]. Proponents believe that a simulated environment helps students practice in a safe environment until competence is attained. Appropriate simulation activities would improve critical reasoning and self-reflection, and lead the way to more self-directed learning [38], which would foster better adjustments to the complex clinical environment and reduce the gap between theory and practice. A qualitative study by Brown [39] in the US using semi-structured interviews among graduating nursing students reaffirmed that high-fidelity simulation can reduce the theory-practice gap and ease the transition to independent clinical practice. None of the current study participants mentioned simulation training and its contributions towards bridging the theory-practice gap. This could be attributed to the fact that simulation training has only recently started gaining momentum. Meanwhile, the UAE currently offers a graduate nurse internship program to novice nurses in its government health care facilities [40], before their assimilation within the UAE health care workforce.

Recent growing evidence indicates that bridging the gap between theory and practice in nursing education is a shared responsibility between academic faculties, registered nurses, and student nurses [14, 15, 35]. There is an urgent need to take a systems view of the multifaceted issue in nursing education. Some other solutions from the literature include a dynamic understanding of the clinical setting, ongoing and timely collaboration with industry clinical partners, a systematic and regular review of the curriculum to ensure inclusion of essential competencies (with exclusion or updating of outdated practices), and incorporation of evidence-based teaching practices to prepare students for the realities of the clinical setting [35].

This study has helped gain awareness about students’ perspectives and concerns about the theory-practice gap in nursing education, and identified possible solutions to tackle the issue and reduce this gap. As a practice-oriented discipline, theory and practice cannot be separated in nursing. Raines [38] suggests using a K-W-L chart (Knowing; Want to know; Learnt) to encourage students to consider what they already know actively, and to prompt them to take active measures to enhance their knowledge and skills. As the body of knowledge grows in nursing education and practice, students need to be more self-directed and active learners to bridge the gap between theory and practice. This would lead to the students’ empowerment to develop personal connections to the learning experience, while taking ownership of their professional growth.

## Conclusion

This study explored the solutions to bridge the theory-practice gap in nursing education in the UAE, from the perspective of the nursing students studying nursing within the country. Emerging categories related to the creation of a clinical culture and the reformation of the nursing curriculum. Future studies can explore solutions to the theory-practice gap among new nursing graduates practicing after their graduation, using mixed-methods approaches for data triangulation and improved rigor.

### Limitations

This study included homogenous group of female nursing students studying in one nursing college. Therefore, the findings of the current study may be transferable when we deal people with similar conditions. Additionally, it will be useful if there is a chance to study theory-practice gap from the perspectives of the newly graduated nurses. Finally, using a qualitative design limits the chance of generalizing the findings of the current study. It will be excellent to study this subject quantitatively.

### Implications in practice

The study has implications for nursing education and practice within the UAE and is imperative for graduating workplace ready professional nurses within the country. The study presents solutions and suggestions to close the gaps in nursing education from the perspective of nursing students. This will be useful to nursing academics, nursing leaders and nursing preceptors by opening their eyes to the students’ needs during clinical training and the ways to bridge the gap between classrooms teaching and the clinical training.

## Data Availability

The data available in form of audiotape and transcripts. All data generated or analysed during this study are included in this article. All the data are saved in a secured computer. The datasets used and/or analysed during the current study are available from the corresponding author on reasonable request.
